# Obesity, antenatal depression, diet and gestational weight gain in a population cohort study

**DOI:** 10.1007/s00737-016-0635-3

**Published:** 2016-05-13

**Authors:** Emma Molyneaux, Lucilla Poston, Mizanur Khondoker, Louise M. Howard

**Affiliations:** 1 Section of Women’s Mental Health, Institute of Psychiatry, Psychology & Neuroscience, King’s College London, PO31 De Crespigny Park, London, SE5 8AF UK; 2 Division of Women’s Health, King’s College London, London, SE1 7EH UK; 3 Department of Applied Health Research, University College London, London, WC1E 7HB UK

**Keywords:** Obesity, Depression, ALSPAC, Pregnancy, Diet

## Abstract

**Purpose:**

The aims of this paper are to examine: (1) the relationship between high pre-pregnancy BMI and antenatal depression; (2) whether BMI and antenatal depression interact to predict diet and gestational weight gain (GWG).

**Methods:**

Data came from the Avon Longitudinal Study of Parents and Children (ALSPAC). Underweight women were excluded. Pre-pregnancy BMI was self-reported and antenatal depression was assessed using the Edinburgh Postnatal Depression Scale at 18 and 32 weeks’ gestation to identify persistently elevated depressive symptoms (EPDS>12). Dietary patterns were calculated from food frequency questionnaires at 32 weeks’ gestation. GWG was categorised using the USA Institute of Medicine guidelines.

**Results:**

This study included 13,314 pregnant women. Obese women had significantly higher odds of antenatal depression than normal weight controls after adjusting for socio-demographics and health behaviours (aOR 1.39, 95%CI 1.05–1.84). Every unit increase in pre-pregnancy BMI was associated with approximately 3% higher odds of antenatal depression (aOR 1.03, 95%CI 1.01-1.05). Antenatal depression was not meaningfully associated with dietary patterns after adjusting for confounders and was not associated with inadequate or excessive GWG. There was no evidence for an interaction of depression and BMI on either diet or GWG.

**Conclusions:**

Healthcare professionals should be aware of the dose-response relationship between high pre-pregnancy BMI and antenatal depression.

**Electronic supplementary material:**

The online version of this article (doi:10.1007/s00737-016-0635-3) contains supplementary material, which is available to authorized users.

## Introduction

The prevalence of obesity is rising globally, and approximately 20% of women in the UK and USA are now estimated to be obese when they become pregnant (Heslehurst et al. 2007; Fisher et al. 2013). The physical health risks associated with obesity in pregnancy, including preeclampsia and gestational diabetes (e.g. Sebire et al. 2001), are well established but the relationship between obesity and maternal mental health has been largely neglected, as has the potential influence of mental health on the health behaviours and physical health of obese pregnant women.

A recent systematic review and meta-analysis (Molyneaux et al. 2014) found that women who were obese when they became pregnant were more likely to experience antenatal depression than women of normal weight (OR 1.43, 95 %CI 1.27–1.61). However, this meta-analysis pooled unadjusted odds ratios and the association may be explained by confounding influences. Laraia et al. (2009) had previously reported that the association between high pre-pregnancy body mass index (BMI) and symptoms of antenatal depression remained significant after adjusting for socio-demographic factors, but these authors did not examine any other potential confounders, such as health behaviours. In addition, some studies of non-pregnant adults have suggested that obesity may be more strongly associated with depression among those with higher socio-economic status (SES) compared to those with lower SES (Moore et al. 1962; Simon et al. 2006), but this association has not been explored in pregnant women.

Antenatal depression has been associated with poor diet and both inadequate and excessive gestational weight gain (GWG), but the literature is very inconsistent (Hurley et al. 2005; Rasmussen and Yaktine 2009; Fowles et al. 2011; Hartley et al. 2015; Kapadia et al. 2015). It has been suggested that in non-pregnant adults the relationship between depression and weight gain may vary according to BMI (Murphy et al. 2009) but the influence of BMI on the relationship between antenatal depression and weight gain in pregnancy is underexplored. One study on this topic (Bodnar et al. 2009) found that antenatal depression and excessive GWG were only associated among women who had high BMI at the start of pregnancy, but this finding requires replication.

In this study, we examined the relationships between pre-pregnancy obesity, antenatal depression, diet and GWG using data from the Avon Longitudinal Study of Parents and Children (ALSPAC). First, the relationship between pre-pregnancy BMI and antenatal depression was investigated; including adjustment for confounders and examination of the interaction of BMI category and SES on the risk of antenatal depression. The associations between antenatal depression and (1) dietary patterns during pregnancy; and (2) GWG were then examined, and the interactions of antenatal depression and pre-pregnancy BMI on these outcomes were tested.

## Materials and methods

### Study population

This study used data from the ALSPAC prospective population cohort study which recruited women in early pregnancy. Detailed descriptions of the ALSPAC study design and cohort profile have been published (Golding et al. 2001; Boyd et al. 2012). Please note that the study website contains details of all the data that is available through a fully searchable data dictionary (http://www.bris.ac.uk/alspac/researchers/data-access/data-dictionary/). All pregnant women living in the former Avon Health Authority, South West England, with due dates between 1st April 1991 and 31st December 1992 were eligible to participate. The ALSPAC study initially enrolled 14,541 women. An estimated 85–90% of the women approached agreed to participate (O’Connor et al. 2003) and the sample included approximately 72% of potentially eligible women (Boyd et al. 2012). Ethical approval was obtained from the ALSPAC ethics committee and the local research ethics committees. No additional ethical approval was required for these analyses.

Women in the ALSPAC cohort who experienced pregnancy or neonatal loss (*n* = 652) were excluded from the sample for these analyses, as were women who were underweight at the start of pregnancy (based on self-reported BMI<18.5 kg/m^2^; *n* = 575) who were not the focus of this study. 91.6 % of women in the original cohort were included in the current study.

### Measures

#### Body mass index

In the ALSPAC study, women self-reported their height and retrospectively self-reported their pre-pregnancy weight as part of a postal questionnaire sent to women after enrolment. This was used to calculate pre-pregnancy BMI, classified using the World Health Organisation (2000) categories of normal weight (BMI 18.5–25 kg/m^2^), overweight (BMI 25–30 kg/m^2^) and obese (BMI ≥30 kg/m^2^).

#### Antenatal depression

Antenatal depression was assessed using the Edinburgh Postnatal Depression Scale (Cox et al. 1987). The EPDS was included in postal questionnaires sent to women at approximately 18 and 32 weeks’ gestation. A cut-off of >12 was used, previously validated in the third trimester of pregnancy (Murray and Cox 1990). For this study, cases of antenatal depression were defined as those scoring >12 on the EPDS at both 18 and 32 weeks’ gestation (i.e. persistently high levels of depressive symptoms). Repeated assessment with the EPDS has been shown to have a higher positive predictive value for depression (based on a clinical diagnostic interview) than a single EPDS assessment (Nyklíček et al. 2004).

#### Dietary patterns

Diet was assessed using a self-reported food frequency questionnaire at approximately 32 weeks’ gestation. Participants indicated how many times each week they currently ate each of 43 food items or groups, representing the core food types in the British diet at that time (see Rogers and Emmett (1998) for full details). Example items include sausages, burgers, rice (boiled), and cabbage, Brussels sprouts, kale and other green leafy vegetables. Portion size was not measured to avoid overburdening the participants. Completed questionnaires were received from 12,436 women. Principal component analysis was performed in a previous study by Northstone et al. (2008) to derive dietary patterns from the food frequency questionnaire and five components were identified as the best representation of the data. The components were classified by Northstone et al. as five dietary patterns based on highly loading food items: health conscious (characterised by consumption of salad, fruit, rice, fish, white meat, non-white bread), traditional (e.g. vegetables, red meat, poultry, potatoes), processed (e.g. pizza, sausages/burgers, chips), confectionary (e.g. biscuits, puddings, cake/buns, sweets), and vegetarian (e.g. soya, tofu, pulses; inverse associations with red meat and poultry). Northstone et al. (2008) calculated standardised scores for each of the five dietary patterns for all participants, with higher scores representing greater similarity to that dietary pattern.

#### Gestational weight gain

All weight measurements during pregnancy were extracted from obstetric records by six trained research midwives. Fraser et al. (2010) used these measurements to predict weight gain across the entire pregnancy using random effects statistical modelling. Total GWG was then categorised by the ALSPAC study team based on the USA Institute of Medicine guidelines which provide recommended ranges for GWG based on the mother’s pre-pregnancy BMI category (Rasmussen and Yaktine 2009). The weight gain recommendations are 11.5–16 kg for women who are normal weight at the start of pregnancy, 7–11.5 kg for women who are overweight at the start of pregnancy and 5–9 kg for women who are obese at the start of pregnancy. Using these recommendations, weight gain was classified as inadequate (below the recommended range), recommended (within the recommended range) or excessive (above the recommended range).

#### Covariates

Socio-demographic factors (age, ethnicity, marital status, occupation, highest educational level, parity, singleton or multiple pregnancy, stressful life events during pregnancy and social support during pregnancy) and early pregnancy health behaviours (alcohol consumption, tobacco smoking, drug use, and physical activity) were included as covariates in this study. More detail on the measurement and coding of these variables is given in Online Resource 1.

### Statistical analyses

#### Missing data and multiple imputation

Over half (56.2%; *n* = 8165) of participants had missing data for at least one of the analysis variables but only 20.5% (*n* = 2981) of participants had missing data for more than five variables. All variables had some missing data; the proportion of missing data per variable ranged from 0.5 to 31.2% (see Online Resource 2 for individual variable information). The overall proportion of missing data for all analysis variables was 16.3%. Conducting complete case analyses would therefore cause loss of statistical power and potentially lead to biased estimates and incorrect inferences. Patterns of missing data were examined and the assumption of missing at random was found to be plausible (see Online Resource 3). Missing values were therefore imputed using multiple imputation by chained equations (Van Buuren and Oudshoorn 1999) with the *ice* command in Stata 12 (Royston 2005).

A full list of the variables in the multiple imputation model is given in Online Resource 2. The imputation model included all analysis variables, relevant interaction terms (e.g. BMI × depression) and related auxiliary variables. The inclusion of auxiliary variables increases the plausibility of missing at random and can improve the prediction of missing values (Sterne et al. 2009). Prior to imputation, regression assumptions were checked. Linear regression was used to impute normally distributed continuous variables, logistic regression was used for dichotomous variables and ordered logistic regression was used for ordered categorical variables. Predictive mean matching, a non-parametric imputation technique, was used to impute semi-continuous variables such as EPDS score. Sixty-five imputed datasets were produced, to fulfil the recommendation of more imputations than the percentage of incomplete cases (Bodner 2008). After imputation, the values of the imputed and observed datasets were compared and found to be broadly consistent (see Online Resource 4).

Using Stata 12, the statistical analyses described below were performed on each imputed dataset separately and combined using Rubin’s rules (Rubin 1987) which take into account the uncertainty of imputed data when calculating overall standard errors. The main unadjusted and adjusted analyses were also repeated using complete case analysis to examine the influence of multiple imputation on findings.

#### Main analyses

Characteristics of the sample were examined. Logistic regression was used to calculate the relationships between pre-pregnancy overweight or obesity and risk of antenatal depression, with women who were normal weight at the start of pregnancy as the control group. The effects of adjusting for socio-demographic factors and health behaviours were examined in two multiple logistic regression models: the first including socio-demographic factors only and the second additionally including health behaviours. Singleton or multiple pregnancy, social support in pregnancy and stressful life events in pregnancy were not included as potential confounders in these analyses as these variables could not have caused high pre-pregnancy BMI. All other covariates were included in all analyses. The interaction of pre-pregnancy BMI category and SES on antenatal depression was tested (SES based on occupation: professional, managerial or technical vs. manual or unskilled). The unadjusted and fully adjusted analyses were also repeated using pre-pregnancy BMI as a continuous predictor.

Linear regression was used to examine the associations between antenatal depression and dietary patterns in the third trimester. The interaction of antenatal depression and pre-pregnancy BMI category on dietary pattern scores was tested. Multiple logistic regression models were used to examine the association between antenatal depression and dietary patterns adjusted for (1) socio-demographic factors and (2) socio-demographic factors, health behaviours and BMI.

Finally, the relationship between antenatal depression and risk of inadequate or excessive GWG was examined using multinomial logistic regression, with recommended GWG as the base outcome. Each analysis compared the odds of (1) inadequate and (2) excessive GWG with the odds of recommended GWG, for women with antenatal depression compared to those without antenatal depression. The interaction of antenatal depression and pre-pregnancy BMI category on GWG was tested. The effect of adjusting for confounders was examined in two adjusted models, the first controlling for socio-demographic factors only and the second additionally controlling for health behaviours and BMI.

## Results

In total, 14,541 women were recruited into the ALSPAC cohort during pregnancy; 13,314 of these women were included in the current study following multiple imputation of missing data and exclusion of women with pregnancy or neonatal loss (*n* = 652) as well as women who were underweight at the beginning of pregnancy (*n* = 575).

Sample characteristics are given in Table 1. The majority of women in the sample were aged 25–34 (66.3%), of white ethnicity (97.3%) and married (74.4%). Just under half of the women were nulliparous (44.9%) and 98.7% had singleton pregnancies. Approximately two thirds of the women (64.6%) had no educational qualifications beyond the Certificate of Secondary Education or O levels (exams taken at the age of 16). One third (33.5%) were currently or previously employed in professional, managerial or technical occupations, half (51.1%) had routine non-manual or skilled manual occupations and the remaining 15.4% had partly or unskilled manual occupations.

**Table 1:** Characteristics of the sample and associations with pre-pregnancy BMI category and antenatal depression status

		Overall sample	Pre-pregnancy BMI category				Antenatal depression status		
			Normal weight	Overweight	Obese	*p* value	Not depressed	Depressed	*p* value
Total sample; %			77.8	16.1	6.1	–	92.1	7.9	–
Socio-demographic factors									
Age; %	<20	4.5	84.3	12.2	3.5	<0.001	82.4	17.6	<0.001
	20–24	19.2	75.6	16.8	7.6		88.1	11.9	
	25–34	66.3	78.2	16.1	5.7		93.7	6.3	
	35–39	8.8	77.4	15.6	7.0		93.2	6.8	
	40+	1.2	73.8	20.5	5.8		93.1	6.9	
Ethnicity; %	White	97.3	77.8	16.1	6.1	0.292	92.3	7.7	<0.001
	Other	2.7	80.6	14.2	5.2		83.6	16.4	
Marital status; %	Unmarried	25.6	80.3	14.2	5.5	0.001	86.3	13.7	<0.001
	Married	74.4	77.0	16.7	6.3		94.1	5.9	
Parity; %	Nulliparous	44.9	80.8	14.5	4.7	<0.001	92.9	7.1	0.004
	Parous	55.1	75.4	17.4	7.2		91.4	8.6	
Pregnancy size; %	Singleton	98.7	77.8	16.1	6.1	0.309	92.1	7.9	0.225
	Multiple	1.3	81.4	13.2	5.4		89.4	10.6	
Education; %	Degree	12.4	87.4	9.9	2.8	<0.001	95.1	4.9	<0.001
	A level	22.0	80.8	14.9	4.3		94.5	5.5	
	O level	34.5	77.6	16.5	5.9		92.3	7.7	
	CSE^a^/vocational	30.1	72.2	18.9	8.9		88.9	11.1	
Occupation^b^; %	I or II	33.5	81.7	13.9	4.4	<0.001	94.3	5.7	<0.001
	IIa or IIIb	51.1	76.8	16.7	6.5		92.0	8.0	
	IV or V	15.4	72.8	18.9	8.3		87.5	12.5	
Social support; %	Low	35.7	75.8	17.0	7.3	<0.001	85.0	15.0	<0.001
	Medium	35.0	79.1	15.3	5.7		94.7	5.3	
	High	29.4	78.9	16.0	5.1		97.6	2.5	
Stressful life events; %	0–2	38.7	78.3	16.0	5.7	0.602	97.1	2.9	<0.001
	3–5	40.1	77.4	16.4	6.2		92.8	7.2	
	6+	21.2	77.9	15.6	6.5		81.6	18.4	
Health behaviours									
Alcohol consumption; %	None	45.4	76.5	16.7	6.8	0.003	92.2	7.8	0.006
	<1 glass daily	52.7	79.0	15.5	5.5		92.3	7.8	
	≥1 glass daily	2.0	76.5	18.4	5.1		84.4	15.6	
Smoking; %	No	74.8	77.9	16.0	6.1	0.990	93.9	6.1	<0.001
	Yes	25.2	77.8	16.2	6.0		86.6	13.4	
Drug use; %	No	97.3	77.5	16.3	6.2	<0.001	92.5	7.6	<0.001
	Yes	2.7	90.1	8.2	1.7		78.9	21.1	
Physical activity; %	None	15.4	74.2	17.8	8.0	<0.001	89.8	10.2	0.006
	≤1 h/week	28.1	76.9	16.6	6.5		92.6	7.4	
	≥2 h/week	56.5	79.3	15.4	5.4		92.4	7.6	

^a^ *CSE* Certificate of Secondary Education

^b^I or II: professional, managerial or technical; IIIa or IIIb: routine non-manual or skilled manual; IV or V: partly skilled or unskilled manual (Based on Office of Population Censuses and Surveys occupational classifications, 1991)

At 18 weeks’ gestation, the median score on the EPDS was 6 (interquartile range 3–10); 14.3 % of women scored >12 at this time. At 32 weeks’ gestation, the median score on the EPDS was 7 (interquartile range 3–11) and 15.7 % of women scored >12. Overall, 7.9 % of the study sample had persistently elevated symptoms of depression (EPDS>12 at both 18 and 32 weeks’ gestation) and were defined as having antenatal depression for this study. Antenatal depression was most prevalent among women who were younger, of non-white ethnicity, multiparous and unmarried, as well as those with lower levels of education, manual or unskilled jobs and low social support (see Table 1). Antenatal depression was also more common among women reporting higher alcohol consumption, smoking, illegal drug use or no physical activity during pregnancy.

At the start of pregnancy, 77.8 % of the sample was normal weight, 16.1 % was overweight and 6.1 % was obese. Obesity was less common among women under the age of twenty and more common among women who were married, multiparous, had lower levels of education, manual or unskilled jobs and low social support, as well as among women reporting no alcohol consumption, no illegal drug use or no physical activity (see Table 1).

### Pre-pregnancy obesity and antenatal depression

**Fig. 1:**
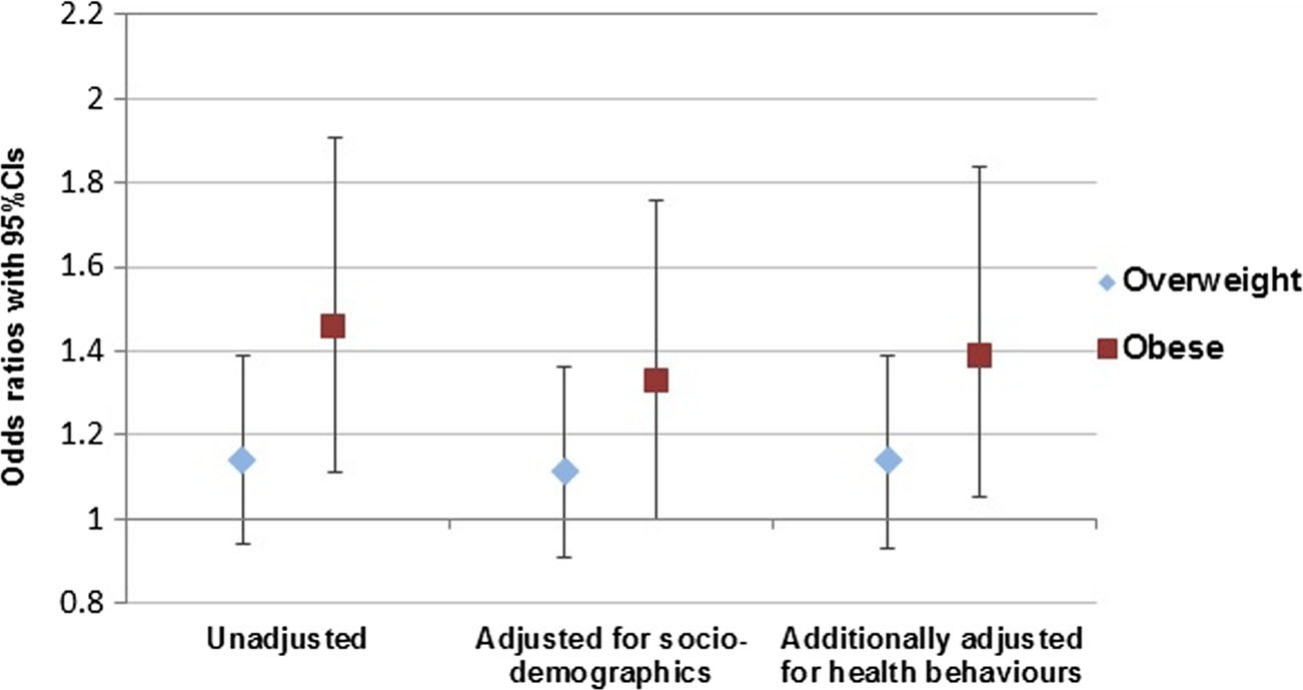
Unadjusted and adjusted associations between pre-pregnancy overweight or obesity and antenatal depression

The prevalence of antenatal depression was lowest among women who were normal weight when they became pregnant (7.6%), intermediate among overweight women (8.5%) and highest among women who were obese when they became pregnant (10.7%). Women who were obese at the start of pregnancy had significantly higher odds of antenatal depression than normal weight women (OR 1.46, 95%CI 1.11 to 1.91, *p* = 0.007) but the association between pre-pregnancy overweight and antenatal depression was not statistically significant (OR 1.14, 95%CI 0.94 to 1.39, *p* = 0.192). After adjusting for socio-demographic factors (age, marital status, educational level, parity, occupation), the association between obesity and antenatal depression reduced in magnitude but remained significant (OR 1.33, 95%CI 1.00 to 1.76, *p* = 0.046). The association between overweight and antenatal depression remained non-significant (OR 1.11, 95%CI 0.91–1.36, *p* = 0.291). Additionally adjusting for health behaviours had little effect on the magnitude of the associations (overweight: OR 1.14, 95%CI 0.93–1.39, *p* = 0.206, obese: OR 1.39, 95%CI 1.05–1.84, *p* = 0.022). The unadjusted and adjusted associations are shown in Fig. 1, with women who were normal weight at the start of pregnancy as the reference group for all analyses. There was no evidence for a significant interaction effect of pre-pregnancy BMI category and SES on risk of antenatal depression (*p* = 0.429 for overweight and *p* = 0.952 for obese).

There was a significant unadjusted association between pre-pregnancy BMI (as a continuous variable) and risk of antenatal depression (OR 1.03, 95%CI 1.01–1.05, *p* = 0.001). This remained significant in the fully adjusted model, with approximately 3% higher odds of antenatal depression for every unit increase in pre-pregnancy BMI (OR 1.03, 95%CI 1.01–1.05, *p* = 0.001).

### Antenatal depression and dietary patterns

As shown in Table 2, women with antenatal depression had significantly lower healthy and traditional dietary pattern scores and significantly higher processed and vegetarian dietary pattern scores than women without high levels of antenatal depression symptoms. There was no evidence for a significant interaction of antenatal depression and pre-pregnancy BMI on dietary patterns (all interaction terms: *p* = 0.086 to *p* = 0.910). After adjusting for socio-demographic confounders, as well as in the fully adjusted model, antenatal depression was significantly associated with higher confectionary dietary pattern scores only (also shown in Table 2).

**Table 2:** Associations between antenatal depression and dietary pattern scores

	Unadjusted		Adjusted for socio-demographic factors		Additionally adjusted for health behaviours and BMI	
	β coefficient (95%CI)	*p* value	β coefficient (95%CI)	*p* value	β coefficient (95%CI)	*p* value
Healthy	−0.32 (−0.39 to −0.25)	<0.001	−0.03 (−0.09 to 0.03)	0.343	0.00 (−0.07 to 0.06)	0.872
Traditional	−0.11 (−0.18 to −0.04)	0.002	−0.04 (−0.12 to 0.03)	0.248	−0.04 (−0.11 to 0.03)	0.287
Processed	0.22 (0.15 to 0.30)	<0.001	0.02 (−0.05 to 0.09)	0.581	0.00 (−0.07 to 0.08)	0.924
Confectionary	0.06 (−0.01 to 0.14)	0.091	0.09 (0.02 to 0.17)	0.017	0.10 (0.02 to 0.17)	0.014
Vegetarian	0.16 (0.09 to 0.23)	<0.001	0.07 (−0.01 to 0.14)	0.084	0.05 (−0.02 to 0.13)	0.177

### Antenatal depression and gestational weight gain

The proportion of women with GWG within the recommended range was very similar among those with and without antenatal depression (27.9 and 28.1% respectively), whilst inadequate GWG was slightly more common among women with antenatal depression than those without (14.0 vs. 12.0% respectively) and excessive GWG was slightly less common (58.1 vs. 59.9% respectively). Women with antenatal depression did not have significantly higher odds of either inadequate or excessive GWG than women without antenatal depression in the unadjusted analyses (inadequate GWG: OR 1.17, 95%CI 0.92–1.50, *p* = 0.198; excessive GWG: OR 0.98, 95%CI 0.83–1.15, *p* = 0.787, compared with recommended GWG as the base outcome). There was also no evidence for an interaction of antenatal depression and pre-pregnancy BMI category on risk of inadequate or excessive GWG (interaction terms: *p* = 0.328 to *p* = 0.906). The association between depression and GWG did not change substantially after adjusting for socio-demographic factors (inadequate GWG: OR 1.06, 95%CI 0.82–1.38, *p* = 0.652; excessive GWG: OR 1.00, 95%CI 0.84–1.19, *p* = 0.970) or in the fully adjusted analyses (inadequate GWG: OR 1.03, 95%CI 0.79–1.34, *p* = 0.844; excessive GWG: OR 0.99, 95%CI 0.82-1.18, *p* = 0.880).

### Complete case analysis

In the complete case analysis, there was a significant unadjusted association between pre-pregnancy obesity and antenatal depression (OR 1.55, 95%CI 1.15–2.10, *p* = 0.004) but the association was not significant for overweight women (OR 1.15, 95%CI 0.92–1.42, *p* = 0.214), both compared with normal weight controls (based on 9915 women with complete data for these variables). Neither association was significant in the fully adjusted complete case analyses, which included 7464 participants (overweight OR 1.07, 95%CI 0.82–1.40, *p* = 0.624; obese OR 1.32, 95%CI 0.88–1.96, *p* = 0.177). The association between continuous pre-pregnancy BMI and antenatal depression was statistically significant in both the unadjusted (OR 1.04, 95%CI 1.02–1.06, *p* < 0.001) and fully adjusted complete case analyses (OR 1.04, 95%CI 1.02–1.07, *p* = 0.002). There were no meaningful differences in findings for the associations of antenatal depression with dietary patterns or GWG between the complete case and imputed analyses (results of complete case analyses given in Online Resource 5).

## Discussion

Women who were obese when they became pregnant had nearly 40% higher odds of antenatal depression (defined as persistent high levels of depressive symptoms in pregnancy) than women who were normal weight, after adjusting for socio-demographic factors and health behaviours. There was also evidence for a small but statistically significant increase in the odds of antenatal depression for each unit increase in pre-pregnancy BMI. Hence there is a dose-response relationship between BMI and depression in pregnancy that is not explained by socio-demographic or lifestyle risk factors and health professionals should ensure to ask about depressive symptomatology in obese pregnant women. Women with antenatal depression reported less healthy dietary patterns during the third trimester than women without depression, but these associations were almost entirely accounted for by socio-demographic confounding. Finally, antenatal depression was not associated with either inadequate or excessive GWG, and there was no evidence for an interaction of pre-pregnancy BMI category and antenatal depression status on either dietary patterns or GWG.

The unadjusted association between pre-pregnancy obesity and antenatal depression found in this study was similar to the pooled estimate for this association obtained in a recent systematic review and meta-analysis (Molyneaux et al. 2014). In the current study, adjusting for socio-demographic factors slightly reduced the strength of the association between obesity and antenatal depression but the association remained statistically significant. This corresponds to similar findings of a study by Laraia et al. (2009), which examined the association between pre-pregnancy BMI and symptoms of depression during pregnancy. The current study extended previous research by examining early pregnancy health behaviours, but found no evidence for a role of these variables as confounders or mediators of the association between pre-pregnancy obesity and antenatal depression. Future research on the association between obesity and depression during pregnancy should examine other potential mechanisms such as poor body image, obesity-related stigma and physical health problems (Siegel et al. 2000; Jorm et al. 2003; Friedman et al. 2008; Gavin et al. 2010). There was no evidence in this study for an interaction of pre-pregnancy BMI and SES on the risk of antenatal depression, which has been observed in some studies of non-pregnant adult women (Moore et al. 1962; Simon et al. 2006).

This study also added to the existing literature by examining whether antenatal depression and pre-pregnancy BMI category interacted to predict dietary patterns or GWG, but no evidence of these hypothesised effects were found. In the overall sample, antenatal depression appeared to be a marker of other risk factors for poor diet during pregnancy but was not an important predictor of dietary patterns after adjustment for socio-demographic confounders. A statistically significant adjusted association was found between antenatal depression and higher scores for the confectionary dietary pattern, but the magnitude of this association was minimal and is therefore unlikely to have any public health significance. Antenatal depression was also not associated with inadequate or excessive GWG in this study which is in keeping with some, but not all, previous research (Rasmussen and Yaktine 2009; Hurley et al. 2005; Kapadia et al. 2015). One previous study found that pre-pregnancy BMI influenced the association between antenatal depression and excessive GWG (Bodnar et al. 2009) but no evidence for this was found in our study.

### Strengths and limitations

ALSPAC is a very large population-based cohort study with detailed data collection during pregnancy. The cohort was broadly representative of the population at the time of recruitment (1991–1992), although ethnic minority women were underrepresented, and married or cohabiting women, owner-occupiers and those with a car in their household were overrepresented (Golding et al. 2001). However, there have been considerable changes in the characteristics of the UK population since the early 1990s, including substantial increases in the prevalence of obesity and the proportion of women who are obese at the start of pregnancy (Heslehurst et al. 2007; 2009). The relationship between obesity and depression may be influenced by the prevalence of obesity and other related factors such as stigma or knowledge of the health risks of obesity. The results of this study therefore require replication in a more recent dataset.

Large cohort studies such as ALSPAC often have substantial missing data which reduces statistical power and can lead to bias. The use of multiple imputation to address missing data was therefore an important strength of this study. Multiple imputation leads to unbiased estimates and standard errors based on the assumption that data are missing at random (Van Buuren and Oudshoorn 1999; Janssen et al. 2010), which was found to be plausible in this sample. Complete case analyses gave broadly similar results to the imputed analyses, although obesity was not found to be significantly associated with increased risk of antenatal depression in the fully adjusted complete case analysis. This may be due to lower power in the complete case analysis or due to bias, as women with antenatal depression were more likely to have missing data for the confounding variables, and therefore more likely to be excluded from this complete case analysis.

Although prospective cohort data was used in this study, the directions of causality in the identified associations are not clear. For example, any causal relationship between pre-pregnancy BMI and antenatal depression may occur because previous episodes of depression had led to pre-pregnancy weight gain. There is systematic review evidence from longitudinal studies in non-pregnant adults supporting a bidirectional association between obesity and depression (Luppino et al. 2010), which may also be the case during pregnancy. Similarly, reverse causality or a bidirectional association is possible for any relationship between depression and diet during pregnancy (Bodnar and Wisner 2005).

Measurement bias is a possible limitation of this study as almost all variables were self-reported. For example, self-reported BMI is often underestimated as individuals usually slightly overestimate their height and underestimate their weight (Huber 2007). However, retrospectively self-reported pre-pregnancy BMI has been found to be highly correlated with weight measured at the first antenatal appointment in the ALSPAC sample (Macdonald-Wallis et al. 2010), and there is evidence that depression does not influence the accuracy of self-reported BMI among obese women (Jeffery et al. 2008; White et al. 2009). The use of a screening measure to assess antenatal depression in the ALSPAC cohort limits the conclusions which can be drawn from this study, as women with high levels of depressive symptoms assessed by the EPDS may not meet the diagnostic criteria for major depressive disorder and vice versa. However, diagnostic assessment of depression would not have been feasible given the large sample size and use of postal questionnaires for the majority of data collection. In addition, the identification of women with persistently elevated symptoms of depression, which has been shown to be more predictive of major depressive disorder than a single EPDS elevated score (Nyklíček et al. 2004), was a strength of this study. Finally, residual confounding may occur as a result of inaccurately or incompletely assessed self-reported confounding factors. For example, sedentary time has been found to be associated with depression independent of physical activity (Teychenne et al. 2010), and should be examined in future research on the relationship between obesity and depression.

### Conclusions

Healthcare providers should be aware of the increased risk of antenatal depression (or high levels of depressive symptoms) among women who are obese when they become pregnant. The adjusted association indicated approximately 40% higher odds of antenatal depression for obese women compared with normal weight controls. This association may have public health significance given the high prevalence of both obesity and depression during pregnancy. However, there were no meaningful associations between antenatal depression and dietary patterns after adjusting for socio-demographic confounders and no associations between antenatal depression and increased risk of inadequate or excessive GWG in this sample. Future research is needed to replicate these findings in a more recent cohort and to extend the evidence base to examine the relationships between antenatal depression and adverse pregnancy outcomes (such as preeclampsia or gestational diabetes) among women with high pre-pregnancy BMI.

## Electronic supplementary material

Below is the link to the electronic supplementary material.

ESM 1 Online Resource 1 Assessment of socio-demographic and health behavioural variables (PDF 55 kb)

ESM 2 Online Resource 2 Variables included in the multiple imputation model and proportion of missing data for each variable (PDF 30 kb)

ESM 3 Online Resource 3 Associations between participant characteristics and missing data (PDF 59 kb)

ESM 4 Online Resource 4 Characteristics of the ALSPAC dataset before and after multiple imputation (PDF 19 kb)

ESM 5 Online Resource 5 Complete case analyses for the relationships between probable antenatal depression and diet and GWG (PDF 33 kb)
