# Supplementary material for: Obesity, antenatal depression, diet and gestational weight gain in a population cohort study
Source: Arch Womens Ment Health. 2016 May 13;19(5):899–907. doi: 10.1007/s00737-016-0635-3 (PMC5021737; doi:10.1007/s00737-016-0635-3)
Supplement: Supplementary file 1 — Online Resource 1 Assessment of socio-demographic and health behavioural variables (PDF 55 kb) [file 737_2016_635_MOESM1_ESM.pdf]

Online Resource 1: Assessment of socio-demographic and health behavioural variables

Variable	Method of measurement
**Age**	Maternal age at delivery was calculated based on the mother's self-reported date of birth and the date of the child's birth.
**Ethnicity**	Ethnicity was self-reported at 32 weeks' gestation and classified as "white" or "other ethnicity" owing to the small numbers of women in the sample with non-white ethnicity.
**Marital Status**	Marital status was self-reported at 8 weeks' gestation with the options "never married", "widowed", "divorced", "separated", "1st marriage" or "marriage 2/3". This was coded as a dichotomous variable "married" or "not married"
**Parity**	Parity (defined as the number of previous pregnancies which resulted in a live birth or stillbirth) was calculated at 18 weeks' gestation based on the number of previous pregnancies each participant reported with the number of miscarriages, abortions or terminations that they reported subtracted from this.
**Singleton or multiple pregnancy**	Pregnancy size (i.e. singleton, twin, triplet or quadruplet pregnancy) was obtained from routinely collected data and used as a dichotomous variable in the analyses to indicate singleton or multiple pregnancy, as the proportion of participants with triplet or quadruplet pregnancies was extremely low.
**Educational level**	At 32 weeks' gestation, women were asked to indicate which educational qualifications they had received (e.g. A-Levels, Apprenticeship, State Registered nurse). These answers were coded for analysis as an ordered categorical variable indicating highest education qualification: none, Certificate of Secondary Education or introductory-intermediate vocational qualification (e.g. shorthand typing, apprenticeship, City & Guilds intermediate technical qualification); O level; A level, or degree level. Women who left this question blank were assumed to have no educational qualifications.
**Occupation**	Women reported their current or most recent occupation at 32 weeks' gestation. This was classified using the Office of Population Censuses and Surveys (1991) standard occupational classifications as I (professional), II (managerial or technical), IIIa (routine non-manual), IIIb (skilled manual), IV (partly skilled) and V (unskilled work). This variable was used to indicate socio-economic status for the interaction term (see Statistical Methods).
**Social support**	At 12 weeks' gestation, women were asked about their social support (10 questions, e.g. "My partner provides the emotional support I need", "I believe in moments of difficulty my neighbours would help me") and social network (10 questions, e.g. "Overall, would you say you belong to a close circle of friends", "How many of your family and friends would help you in times of trouble?"). These 20 items were combined to give an overall score for social support (range 6-59) which was used as a continuous variable (given in tertiles in the characteristics tables).
**Stressful life events**	At 18 weeks' gestation, women were asked to indicate which stressful life events they had experienced during their pregnancy so far based on a list of 39 possible items (example life events include: a friend or relative was ill, you had problems at work, your house or car was burgled, your partner was emotionally cruel to you, you were bleeding and thought you might miscarry). This was used as a continuous variable (range 0 to 18) with higher scores indicating more stressful life events during pregnancy.
**Drug use**	At 18 weeks' gestation, women reported whether they had smoked cannabis during the first trimester or taken other drugs (amphetamines, barbiturates, crack, cocaine, heroin, methadone, ecstasy, other). This was coded as a dichotomous variable indicating no drug use or any drug use.
**Alcohol consumption**	At 18 weeks' gestation, women reported their frequency of alcohol consumption in the first trimester (never, <1 glass/week, ≥1 glass/week, 1-2

Variable	Method of measurement
	glasses/day, 3-9 glasses/day, ≥10 glasses/day).
**Smoking**	At 18 weeks' gestation, women were asked whether they smoked cigarettes, cigars, or pipes during the first three months of pregnancy. This was coded as a dichotomous variable indicating whether women smoked or not.
**Physical activity**	At 18 weeks' gestation, women reported how many hours per week they currently spent doing each of the following activities: jogging, aerobics, antenatal exercise, keep fit, yoga, squash, tennis or badminton, swimming, brisk walking, weight training, cycling or other exercise. These answers were pooled into one variable which indicated no physical activity, one hour or less per week, or at least two hours per week.
