# Supplementary material for: Obesity, antenatal depression, diet and gestational weight gain in a population cohort study
Source: Arch Womens Ment Health. 2016 May 13;19(5):899–907. doi: 10.1007/s00737-016-0635-3 (PMC5021737; doi:10.1007/s00737-016-0635-3)
Supplement: Supplementary file 2 — Online Resource 2 Variables included in the multiple imputation model and proportion of missing data for each variable (PDF 30 kb) [file 737_2016_635_MOESM2_ESM.pdf]

<u>Online Resource 2: Variables included in the multiple imputation model and proportion of missing data for each variable</u>

Analysis variables		Auxiliary variables	
**Variable and method of imputation**	**Observed n (%)**	**Variable and method of imputation**	**Observed n (%)**
EPDS at 18 weeks' gestation (pmm)	11,999 (82.5)	Pre-pregnancy smoking (logit)	13,190 (90.7)
EPDS at 32 weeks' gestation (pmm)	11,953 (82.2)	Partner occupation (ologit)	10,904 (75.0)
Pre-pregnancy BMI (pmm)	11,529 (78.6)	First trimester binge drinking (ologit)	12,983 (89.3)
Dietary pattern scores (pmm)	12,040 (82.8)	History of depression (logit)	12,448 (85.6)
GWG (modelled) (ologit)	11,061 (76.1)	History of other psychiatric disorder (logit)	12,448 (85.6)
Age (regress)	13,896 (95.6)	Has a partner (logit)	12,413 (85.4)
Marital status (logit)	13,391 (92.1)	Absolute weight gain (regress)	12,286 (84.5)
Ethnicity (logit)	12,248 (84.2)	Crown Crisp Experiential Inventory, 18 weeks (pmm)	11,419 (78.5)
Parity (ologit)	12,957 (89.1)	Crown Crisp Experiential Inventory, 32 weeks (pmm)	11,292 (77.7)
Pregnancy size (logit)	14,472 (99.5)	Financial difficulties (ologit)	12,011 (82.6)
Education level (ologit)	12,340 (84.9)	Pregnancy or neonatal loss (logit)	14,472 (99.5)
Occupational class (ologit)	9,997 (68.8)	Measured GWG (ologit)	11,061(76.1)
Alcohol consumption (ologit)	13,029 (89.6)		
Hard drug use (logit)	12,931 (88.9)		
Marijuana use (logit)	12,142 (83.5)		
Smoking (logit)	13,190 (90.7)		
Physical activity (ologit)	12,206 (83.9)		
Stressful life events (pmm)	12,031 (82.7)		
Social support score (pmm)	11,423 (78.6)		
Social network score (pmm)	12,083 (83.1)		

Pmm: predictive mean matching (with the model specified to pick from the ten closest values; Morris, White et al. (2014))
