# Supplementary material for: Obesity, antenatal depression, diet and gestational weight gain in a population cohort study
Source: Arch Womens Ment Health. 2016 May 13;19(5):899–907. doi: 10.1007/s00737-016-0635-3 (PMC5021737; doi:10.1007/s00737-016-0635-3)
Supplement: Supplementary file 3 — Online Resource 3 Associations between participant characteristics and missing data (PDF 59 kb) [file 737_2016_635_MOESM3_ESM.pdf]

Online Resource 3: Associations between participant characteristics and missing data

Variable		All observed	Any missing	p value
**Probable antenatal depression, 18 weeks; %**	No	54.7	45.3	<0.001
	Yes	43.7	56.3	
**Probable antenatal depression, 32 weeks; %**	No	54.9	45.1	<0.001
	Yes	44.7	55.3	
**Persistent probable antenatal depression; %**	No	54.7	45.3	0.002
	Yes	49.0	51.0	
**BMI; %**	Underweight	48.0	52.0	<0.001
	Normal weight	55.9	44.1	
	Overweight	56.6	43.4	
	Obese	51.0	49.0	
**GWG; %**	Inadequate	57.1	42.9	0.765
	Recommended	57.3	42.7	
	Excessive	57.9	42.1	
**Dietary patterns; mean (sd)**	Healthy	0.14 (0.97)	-0.15 (1.01)	<0.001
	Traditional	-0.02 (0.97)	0.02 (1.04)	0.0177
	Processed	-0.06 (0.89)	0.07 (1.10)	<0.001
	Confectionary	0.03 (0.96)	-0.03 (1.04)	<0.001
	Vegetarian	-0.04 (0.99)	0.04 (1.01)	<0.001
**Age (years); %**	<20	21.5	78.5	<0.001
	20-24	36.6	63.4	
	25-34	50.2	49.9	
	35-39	47.1	52.9	
	40+	47.9	52.1	
**Ethnicity; %**	White	52.7	47.3	<0.001
	Other	29.3	70.7	
**Marital status; %**	Married	51.7	48.3	<0.001
	Unmarried	33.9	66.1	
**Parity; %**	Nulliparous	54.2	45.8	<0.001
	Parous	45.1	54.9	
**Pregnancy size; %**	Singleton	44.7	55.3	<0.001
	Multiple	0.0	100.0	
**Educational level; %**	Degree	62.8	37.2	<0.001
	A level	59.9	40.1	
	O level	55.1	44.9	
	CSE/vocational	36.8	63.2	
**Occupational classification*; %**	I or II	67.1	33.0	<0.001
	IIIa or IIIb	62.6	37.4	
	IV or V	58.5	41.5	
**Social support; %**	Low	50.3	49.7	<0.001
	Medium	59.6	40.4	
	High	61.0	39.0	
**Stressful life events; %**	0-2	53.6	46.4	<0.001
	3-5	54.7	45.3	
	6+	48.8	51.2	
**Drug use; %**	No	51.4	48.6	<0.001
	Yes	41.1	58.9	
**Alcohol consumption; %**	None	48.1	51.9	<0.001
	<1 glass daily	50.1	49.9	
	1+ glass daily	35.5	64.5	
**Smoking; %**	No	51.7	48.3	<0.001
	Yes	38.5	61.5	

Variable		All observed	Any missing	p value
**Physical activity; %**	None	43.1	56.9	<0.001
	≤1 hour/week	56.4	43.6	
	≥2 hours/week	52.7	47.3	
**Pre-pregnancy smoking; %**	No	52.2	47.8	<0.001
	Yes	40.8	59.2	
**Partner occupational social class; %**	High	60.6	39.4	<0.001
	Low	51.3	48.7	
**First trimester binge drinking; %**	No	49.6	50.4	0.001
	Yes	45.6	54.4	
**History of depression; %**	No	52.2	47.8	<0.001
	Yes	41.6	58.4	
**History of other psychiatric disorder; %**	No	51.2	48.8	0.749
	Yes	52.2	47.8	
**Has a partner; %**	No	17.5	82.5	<0.001
	Yes	51.7	48.3	
**Absolute weight gain** (mean, sd)		12.9 (4.6)	12.1 (4.9)	<0.001⁺
**CCEI**†, 18 weeks (median, IQR)		12 (7-17)	13 (8-19)	<0.001‡
**CCEI**†, 32 weeks (median, IQR)		13 (8-19)	15 (10-21)	<0.001‡
**Financial difficulties** (median, IQR)		1 (0-4)	2 (0-5)	<0.001‡
**Pregnancy or neonatal loss; %**	No	46.1	53.9	<0.001
	Yes	1.1	98.9	
**Measured GWG by IOM guidelines; %**	Inadequate	60.3	39.7	0.038
	Recommended	62.8	37.2	
	Excessive	63.0	37.0	

* I or II: professional, managerial or technical; IIIa or IIIb: routine non-manual or skilled manual; IV or V: partly skilled or unskilled manual

†Crown Crisp Experiential Inventory;

⁺ Based on a t-test;

‡Based on Kruskal-Wallis test
