# Supplementary material for: Obesity, antenatal depression, diet and gestational weight gain in a population cohort study
Source: Arch Womens Ment Health. 2016 May 13;19(5):899–907. doi: 10.1007/s00737-016-0635-3 (PMC5021737; doi:10.1007/s00737-016-0635-3)
Supplement: Supplementary file 4 — Online Resource 4 Characteristics of the ALSPAC dataset before and after multiple imputation (PDF 19 kb) [file 737_2016_635_MOESM4_ESM.pdf]

Online Resource 4: Characteristics of the ALSPAC dataset before and after multiple imputation

Variable		Observed data	Imputed data
**Depression 18 weeks; %**	<13	86.1	85.5
	13+	13.9	14.5
**Depression 32 weeks; %**	<13	84.8	84.0
	13+	15.2	16.0
**Depression both; %**	<13	91.7	92.0
	13+	8.4	8.0
**BMI; %**	Underweight	5.0	5.0
	Normal weight	74.4	73.8
	Overweight	15.1	15.4
	Obese	5.5	5.8
**GWG (modelled); %**	Inadequate	12.6	12.5
	Recommended	28.9	29.5
	Excessive	58.5	58.0
**Dietary patterns ; mean (sd)**	Healthy	0.00 (1.00)	-.03 (1.00)
	Traditional	0.00 (1.00)	-.00 (1.00)
	Processed	0.00 (1.00)	.02 (1.02)
	Confectionary	0.00 (1.00)	-.01 (1.00)
	Vegetarian	0.00 (1.00)	.01 (1.01)
**Age; mean (sd)**		28.0 (5.0)	28.0 (5.0)
**Ethnicity; %**	White	97.4	97.2
	Non white	2.6	2.8
**Parity; %**	Primiparous	44.9	45.0
	Multiparous	55.1	55.0
**Pregnancy size; %**	Single	98.6	98.6
	Multiple	1.4	1.4
**Marital status; %**	Married	74.8	74.0
	Unmarried	25.2	26.0
**Educational level; %**	Degree	13.7	12.2
	A level	23.9	21.9
	O level	36.9	34.4
	CSE/vocational/none	25.4	31.6
**Occupation class; %**	I or II	37.3	33.3
	IIIa or IIIb	50.6	51.1
	IV or V	12.0	15.6
**Social support; %**	Low	33.9	35.8
	Medium	35.4	34.9
	High	30.6	29.3
**Stressful life events; %**	0-2	38.8	38.2
	3-5	40.1	40.1
	6+	21.1	21.7
**Alcohol consumption; %**	None	45.4	45.5
	<1 glass daily	52.7	52.6
	1+ glass daily	1.9	2.0
**Smoking; %**	No	74.9	74.2
	Yes	25.2	25.8
**Hard drug use; %**	No	99.5	99.5
	Yes	0.5	0.5
**Marijuana use; %**	No	97.4	97.3
	Yes	2.6	2.7

Variable		Observed data	Imputed data
**Physical activity; %**	None	15.5	15.7
	Low	27.9	28.0
	Moderate-high	56.6	56.3

* I or II: professional, managerial or technical; IIIa or IIIb: routine non-manual or skilled manual; IV or V: partly skilled or unskilled manual (Based on Office of Population Censuses and Surveys occupational classifications, 1991)
