# Supplementary material for: Obesity, antenatal depression, diet and gestational weight gain in a population cohort study
Source: Arch Womens Ment Health. 2016 May 13;19(5):899–907. doi: 10.1007/s00737-016-0635-3 (PMC5021737; doi:10.1007/s00737-016-0635-3)
Supplement: Supplementary file 5 — Online Resource 5 Complete case analyses for the relationships between probable antenatal depression and diet and GWG (PDF 33 kb) [file 737_2016_635_MOESM5_ESM.pdf]

Online Resource 5: Complete case analyses for the relationships between probable antenatal depression and diet and GWG

Probable antenatal depression was associated with all dietary patterns in the unadjusted complete case analysis (see Table 1) including 11,158 participants. Women with probable antenatal depression had significantly lower healthy and traditional dietary pattern scores and significantly higher processed, confectionary and vegetarian dietary pattern scores. After adjusting for confounders, only the association between probable antenatal depression and confectionary diet score remained statistically significant (6,950 participants included in the adjusted analyses), see Table 1.

Table 1: Association between probable antenatal depression and dietary pattern scores (complete case analyses)

	**Unadjusted**		**Fully adjusted**	
	**β coefficient (95%CI)**	**p value**	**β coefficient (95%CI)**	**p value**
**Healthy**	-0.32 (-0.39 to -0.24)	<0.001	-0.05 (-0.13 to 0.04)	0.270
**Traditional**	-0.12 (-0.19 to -0.05)	0.002	0.00 (-0.10 to 0.10)	0.987
**Processed**	0.19 (0.12 to 0.27)	<0.001	0.05 (-0.04 to 0.13)	0.301
**Confectionary**	0.09 (0.01 to 0.16)	0.019	0.12 (0.02 to 0.22)	0.021
**Vegetarian**	0.16 (0.08 to 0.23)	<0.001	0.06 (-0.05 to 0.16)	0.292

There were no significant associations between probable antenatal depression and GWG in the unadjusted analyses (including 9,691 participants), although there was a trend for greater risk of inadequate GWG among women with probable antenatal depression (inadequate GWG: OR 1.25, 95%CI 0.96-1.64, p=0.098; excessive GWG: OR 0.98, 95%CI 0.81-1.18, p=0.810). Both associations were non-significant in the fully adjusted analyses, including 6,436 participants (inadequate GWG: OR 1.29, 95%CI 0.89-1.87, p=0.181; excessive GWG: OR 1.14, 95%CI 0.88-1.49, p=0.321).
